# Long-term declines in chlorophyll *a* and variable phenology revealed by a 60-year estuarine plankton time series

**DOI:** 10.1073/pnas.2311086121

**Published:** 2024-05-13

**Authors:** Patricia S. Thibodeau, Gavino Puggioni, Jacob Strock, David G. Borkman, Tatiana A. Rynearson

**Affiliations:** ^a^Graduate School of Oceanography, University of Rhode Island, Narragansett, RI 02882; ^b^Department of Computer Science and Statistics, University of Rhode Island, Kingston, RI 02881; ^c^Rhode Island Department of Environmental Management, Office of Water Resources–Shellfish, Providence, RI 02908

**Keywords:** phytoplankton, chlorophyll *a*, nutrients, dynamic linear models, Narragansett Bay

## Abstract

Long-term ecological time series provide a unique perspective on the emergent properties of ecosystems. In aquatic systems, phytoplankton form the base of the food web and their biomass, measured as the concentration of the photosynthetic pigment chlorophyll *a* (chl *a*), is an indicator of ecosystem quality. We analyzed temporal trends in chl *a* from the Long-Term Plankton Time Series in Narragansett Bay, Rhode Island, USA, a temperate estuary experiencing long-term warming and changing anthropogenic nutrient inputs. Dynamic linear models were used to impute and model environmental variables (1959 to 2019) and chl *a* concentrations (1968 to 2019). A long-term chl *a* decrease was observed with an average decline in the cumulative annual chl *a* concentration of 49% and a marked decline of 57% in winter-spring bloom magnitude. The long-term decline in chl *a* concentration was directly and indirectly associated with multiple environmental factors that are impacted by climate change (e.g., warming temperatures, water column stratification, reduced nutrient concentrations) indicating the importance of accounting for regional climate change effects in ecosystem-based management. Analysis of seasonal phenology revealed that the winter–spring bloom occurred earlier, at a rate of 4.9 ± 2.8 d decade^−1^. Finally, the high degree of temporal variation in phytoplankton biomass observed in Narragansett Bay appears common among estuaries, coasts, and open oceans. The commonality among these marine ecosystems highlights the need to maintain a robust set of phytoplankton time series in the coming decades to improve signal-to-noise ratios and identify trends in these highly variable environments.

## Significance

Phytoplankton are the main primary producers in marine environments and their biomass can be used to indicate environmental quality and identify long-term climate-related trends. We analyzed a long-term time series from Narragansett Bay, RI, and found that phytoplankton biomass declined by 49% from 1968 to 2019. The intensity of winter–spring blooms, or biomass peaks that fuel coastal ecosystems, decreased over time and occurred earlier, about 5 d earlier each decade. Shifts in phytoplankton biomass were associated with changes in nutrients, temperature, and salinity. High levels of phytoplankton biomass variation in Narragansett Bay and other time series around the globe highlight the need for longer time series to identify trends from noisy datasets.

Phytoplankton are the main primary producers in marine environments (1, 2). Their biomass can be used as a key indicator in aquatic ecosystems because its magnitude results from growth in response to environmental conditions (e.g., nutrients and light) and mortality due to predation and disease (3–5). Imbalances between the daily rates of asexual division and equally rapid rates of mortality (6) lead to phytoplankton biomass fluctuations that occur over periods of days to months (e.g., refs. 7 and 8) and that influence water quality, biogeochemical cycling, and ecosystem function (9–11). Disentangling daily, seasonal, and annual variation from long-term trends in phytoplankton biomass requires extensive and often high-resolution ecosystem monitoring (12). However, current monitoring programs are frequently shorter than the time of emergence, or the time at which the signal of climate change emerges from the noise of natural climate variability (13). Here, we utilize a 60-y plankton time series, one of the longest of its kind globally, in a warming temperate estuary (14) to examine long-term changes in phytoplankton biomass related to nutrients, seasonality, and bloom phenology.

Phytoplankton biomass is commonly assessed by determining the concentration of the photosynthetic pigment chlorophyll *a* (chl *a*) (15, 16). Over extended time periods (>10 y), chl *a* is an informative tool for determining the effects of climate change and anthropogenic inputs within an ecosystem (17) including shifts in nutrient loading, food webs, and carbon export (e.g., refs. 14, 18, and 19). Longer chl *a* time series also reveal the complexity of marine ecosystems in their responses to climate change (17, 20, 21) and illustrate dynamics that are not always evident in short-term ecosystem studies. For example, although a recent 15-y time series in Long Island Sound, USA, showed chl *a* concentrations increasing over time, the inclusion of intermittent historical samples suggests no long-term trend (22), emphasizing the importance of highly resolved chl *a* records for examining long-term ecosystem change in aquatic environments.

Phenology, the seasonal timing of life history events, represents another important indicator of ecosystem response to climate change, particularly within a long-term context. Shifts in phenology can lead to trophic mismatches and significantly impact local food webs and ecosystem function (23–25). Importantly, the magnitude and direction of phenological shifts in marine organisms in response to climate change are unresolved. For example, a meta-analysis of phenology across a broad range of benthic and planktonic marine organisms revealed advances in both spring and summer phenology of about 4 d decade^−1^ over the global ocean (26). However, regional responses can differ from this global trend (23, 25). For example, despite long-term regional warming in the Gulf of Maine, spring phytoplankton phenology is occurring later in the year, not earlier (27). These conflicting results argue for additional regional investigations of phenological shifts in localized ecosystems, particularly in coastal regions where local anthropogenic activities, such as nutrient loading, interact with larger-scale impacts of climate change, like increasing water temperature (22, 28).

**Fig. 1.**
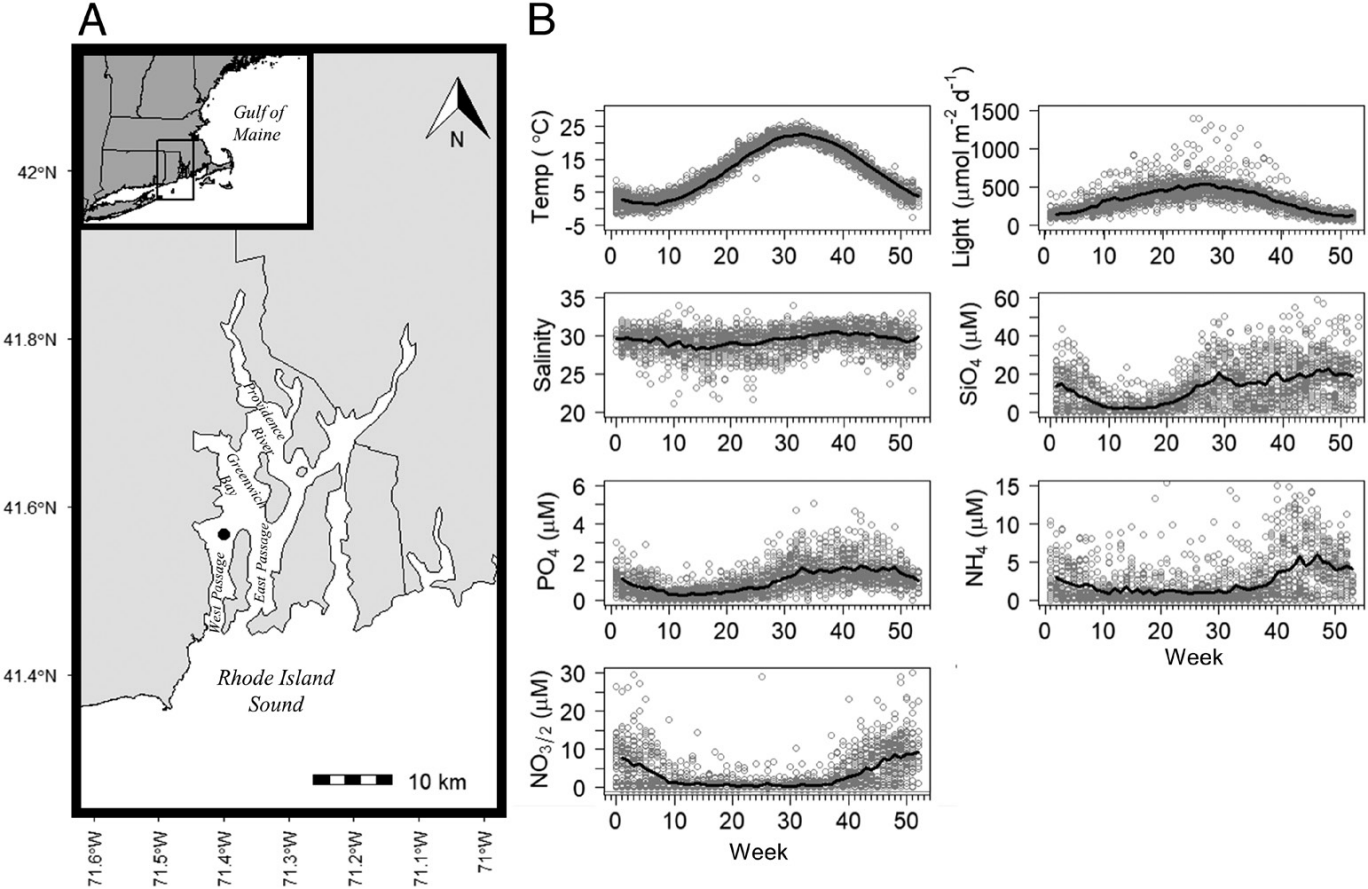
(*A*) Map showing the Narragansett Bay Long-Term Plankton Time Series sampling site in Narragansett Bay, Rhode Island USA. (*B*) Subplots represent weekly data (open circles) and time series mean (black line) for the following parameters; temperature (1959 to 2019), light (1959 to 2019), salinity (1959 to 2019), silicate (SiO_4_, 1959 to 2019), phosphate (PO_4_, 1959 to 2019), ammonium (NH_4_, 1972 to 2019), and nitrate/nitrite (NO_3/2_, 1959 to 2019). Note: outlier values were removed (n < 10) to better illustrate seasonal signal. The complete data are reported in *SI Appendix*, Figs. S2 and S3.

The Narragansett Bay Long-Term Plankton Time Series (NBPTS) provides a unique 60-y (1959 to 2019) perspective on the effects of global change on long-term and phenologic patterns in phytoplankton biomass. Narragansett Bay (NBay) is a coastal estuary connected to the US Northeast Shelf and Northwest Atlantic (Fig. 1*A*) whose production has important implications for fisheries and human use along the coastal US (14, 29). NBay is a highly seasonal system experiencing long-term (1950 to 2015) warming waters and more recently, shifts in anthropogenic nutrient loading through upgrades in wastewater treatment (30–33). Prior analyses of the partial NBPTS chl *a* dataset indicated declines of annual and seasonal means using linear regression (14, 34, 35), but no formal time-series analysis has been conducted on the entire 60-y NBPTS to test hypotheses regarding either the influence of environmental parameters on changing chl *a* concentrations or long-term shifts in phenology.

Here, we digitized historical files (pre-1997) and generated a complete, quality controlled, and harmonized dataset of long-term chl *a* and nutrient concentrations. We used time series analysis to interpolate and model both chl *a* concentrations and a set of environmental parameters to examine the mechanisms underpinning declines in chl *a* concentration. Imputed chl *a* data were then used to obtain and examine seasonal phenology metrics. Due to its broad seasonality and long-term warming, NBay is a model ecosystem to examine estuarine phytoplankton dynamics. Results from this study give important long-term context for bottom–up drivers of variability in an estuary that provides key ecosystem goods and services for the nearly two million people who inhabit its watershed (36).

## Results

### Long-term Trends of Chlorophyll *a* Concentration and Environmental Parameters.

**Fig. 2.**
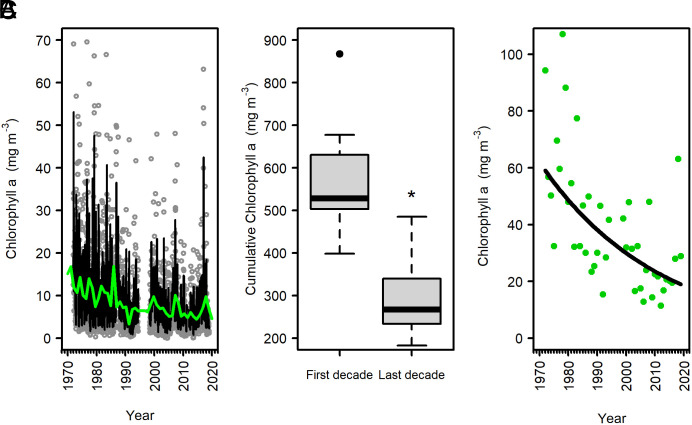
Long-term trends of chlorophyll *a* (chl *a*) concentration from the Narragansett Bay Long-Term Plankton Time Series (1972 to 2019). (*A*) Weekly chl *a* data (circles) with the dynamic linear model (DLM) (black line) and the annual mean of the seasonal component (green line). (*B*) Boxplot of cumulative annual chl *a* values for the first complete decade (1973 to 1982) and last complete decade (2009 to 2019, see *SI Appendix*, *Methods* and Table S2). Mean cumulative chl *a* was significantly different (asterisk) between the first and last decades (*t* test *P* < 0.001, *SI Appendix*, Table S2). (*C*) Annual maximum chl *a* concentrations with the log-transformed model fit (black line, log(x) = −0.02x + 51.75, R^2^ = 0.39; *P* < 0.001). Note: Four data points >75 mg chl m^−3^ shown in *C* were removed from *A* to better illustrate the long-term trend. Data were not sampled the following years: 1995 to 1998 & 2012 (Table 1).

The digitization and quality control of historical and modern time series records yielded 2,013 chl *a* samples (1968 to 2019) and from 1,669 (1972 to 2019, NH_4_) to 2,047 (PO_4_) nutrient samples (1959 to 2019) (Fig. 1*B* and Table 1). Chl *a* concentration in NBay from 1968 to 2019 ranged across five orders of magnitude (0.05 to 107 mg chl m^−3^, *SI Appendix*, Table S1) and was characterized by large variation [average annual coefficient of variation (CV) = 88%], reflecting the high degree of seasonality in NBay. There was a long-term decline in chl *a* concentration, with the steepest drop occurring in the early years of the dataset (1970 to 1989, Fig. 2*A*). Seasonal trends of chl *a* concentration mirrored annual patterns with long-term declines in all seasons at similar rates of change (*SI Appendix*, Fig. S1). Overall, when compared to the first decade of the time series, the cumulative annual chl *a* concentration in the most recent decade declined by 49% (t = 5.59, *P* < 0.001, Fig. 2*B* and *SI Appendix*, Table S2). Although chl *a* concentrations decreased over time, the annual CV did not change significantly between the first and last decades of the study (t = 1.51, *P* = 0.15, *SI Appendix*, Table S2). Bloom intensity (i.e., maximum annual chl *a* concentration) also decreased significantly over time (Fig. 2*C*) dropping 57% between the first and last decades of the study (t = 3.9, *P* = 0.001, *SI Appendix*, Table S2). Annual minimum chl *a* concentrations also decreased (log(x) = −0.02x + 52.89, R^2^ = 0.07; *P* = 0.03).

**Table 1.** Synopsis of physical, chemical, and biological data collected by the Narragansett Bay Long-Term Plankton Time Series indicating when sampling occurred each week (shaded squares), when sampling occurred every other week (asterisks), or when no sampling occurred (white squares). Several decades of data were physically obtained and digitized for this analysis (plus signs)

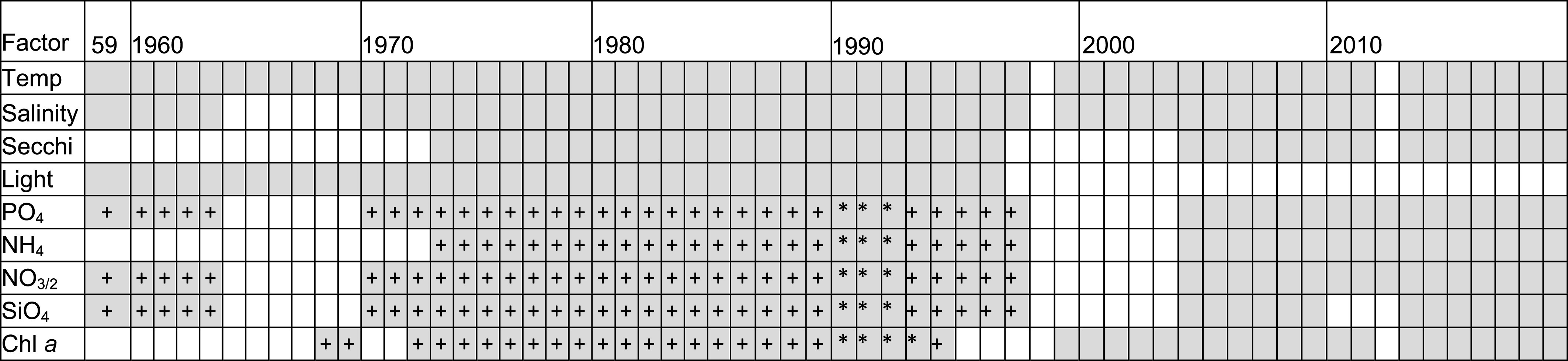

Temp—Temperature (°C), Secchi—Secchi depth, PO_4_—phosphate, NH_4_—ammonium, NO_3/2_—nitrate/nitrite, SiO_4_—silicate, Chl *a*—Chlorophyll *a*.

Like chl *a*, other environmental parameters ranged over several orders of magnitude and had long-term trends (*SI Appendix*, Table S1). Notably, nutrient concentrations exhibited subtle, long-term declines except for SiO_4_ (*SI Appendix*, Fig. S2). Surface water temperature strongly increased over time, particularly after the year 2000 (*SI Appendix*, Fig. S3). Salinity was variable with no long-term directional change. Water column stratification, a function of the difference between surface and bottom water density, decreased, but with large variation around the mean. Both Secchi depth, a proxy for water clarity, and precipitation did not exhibit any long-term trends while light intensity decreased (*SI Appendix*, Table S1 and Fig. S3). The number of cloudy days has increased in NBay possibly explaining the decrease in light we observed (14).

After applying a Bayesian Dynamic Linear Model to interpolate missing values in the dataset, we examined how environmental parameters were related to both short-term (0 to 3-wk lags) and long-term (annual, decadal) fluctuations in chl *a* concentrations from 1970 to 2019. The best-fit seasonal autoregressive integrated moving average with exogenous variable(s) (SARIMAX) model was (2,0,1) × (0,1,1)_52_ with several significant environmental parameters across the 100 SARIMAX model iterations and generally very small deviations in the Monte Carlo (MC) means (Table 2). Mean coefficients of the SARIMAX model indicated that higher chl *a* concentrations were immediately (zero week lag) and significantly related to lower NO_3/2_, NH_4_, and SiO_4_ concentrations as well as lower salinity and temperature and shallower Secchi depth (Table 2). Precipitation, water column stratification, light intensity, and PO_4_ concentration were insignificant parameters, regardless of week lag, and not included in the final SARIMAX model (Table 2).

**Table 2.** Seasonal autoregressive integrated moving average with exogenous variable(s) model, SARIMAX (2,0,1) × (0,1,1)_52_, including the Monte Carlo (MC) mean and standard deviation (SD) for the model parameters, as well as the absolute frequency of times the parameter was significant (*P* < 0.05) out of 100 MC iterations

Parameter	MC-mean	MC-SD	Significance frequency
AR1	1.12	0.03	100
AR2	−0.19	0.03	100
MA1	−0.79	0.02	100
SMA1	−0.95	0.01	100
NH_4_	−0.65	0.04	100
SiO_4_	−0.08	0.01	99
NO_3/2_	−0.23	0.03	100
Secchi	−2.97	0.08	100
Temp	−0.61	0.08	54
Salinity	−2.68	0.16	100
NH_4__lag1	0.06	0.05	1
SiO_4__lag1	0.08	0.01	99
NO_3/2__lag1	−0.18	0.05	87
Salinity_lag1	0.93	0.24	33
NH_4__lag2	0.06	0.04	0
NO_3/2__lag2	0.09	0.03	18
Salinity_lag2	1.38	0.15	100
NO_3/2__lag3	0.23	0.05	100

Model component abbreviations are as follows: AR—Autoregressive component, MA—Moving average, SMA—Seasonal moving average, NH_4_—ammonium, SiO_4_—silicate, NO_3/2_— nitrate/nitrite, Secchi—Secchi depth, Temp—temperature, X_lag1—parameter lagged 1 wk, X_lag2—parameter lagged 2 wk, X_lag3—parameter lagged 3 wk. No lag indicates a zero-week lag.

To examine decadal trends, we utilized Granger causality analysis, which identifies predictive causal relationships between two evolving time series (37, 38), to test the hypothesis that long-term changes of environmental parameters affected long-term changes in chl *a* concentration. We found that NO_3/2_ concentration Granger-caused chl *a* concentration (NO_3/2_ *P* = 0.004). Precipitation also Granger-caused chl *a* concentration (*P* = 0.01). In turn, temperature and water column stratification Granger-caused nutrient concentrations but did not Granger-cause chl *a* concentration (PO_4,_ NO_3/2_, NH_4_, SiO_4_, all *P* < 0.001; chl *a* *P* > 0.05). Light intensity, salinity, and Secchi depth were insignificant parameters (*P* > 0.05).

### Chlorophyll *a* Seasonality and Phenology.

**Fig. 3.**
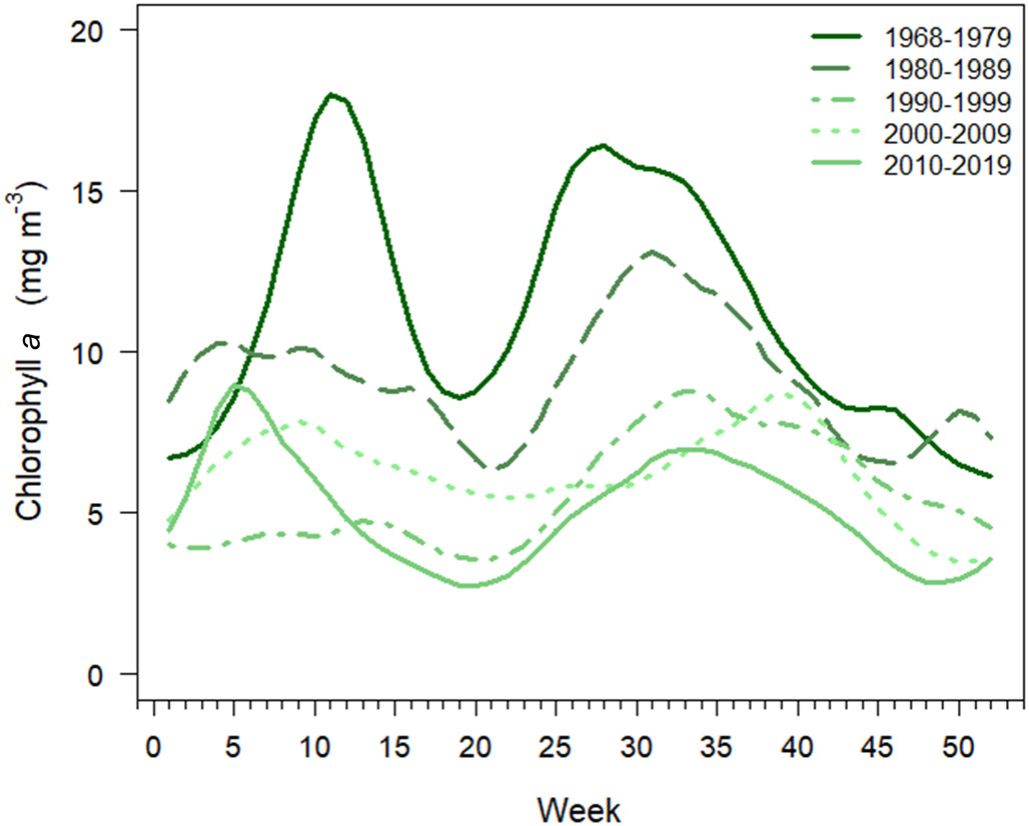
Decadal patterns of weekly chlorophyll *a* (chl *a*) concentrations based on average modeled chl *a*. Decadal patterns were significantly different from each other except the 1990s and the 2010s (*P* < 0.001, K-sample Anderson–Darling test, *SI Appendix*, Table S3).

The chl *a* seasonal cycle was characterized by a bimodal distribution with peaks in concentration occurring both in winter–spring and in summer–fall (Fig. 3). Seasonal patterns in chl *a* concentration showed declines in every decade in both seasons except for a slight increase in winter–spring chl *a* concentration during the last decade (2010 to 2019). Pair-wise comparisons of seasonal chl *a* patterns were significantly different every decade, except between the 1990s and the 2010s (*SI Appendix*, Table S3), despite the marked differences in winter–spring chl *a* distribution between those decades. Nutrient seasonal cycles followed a common pattern of lower concentrations in the spring and summer with increasing concentrations from fall into winter (*SI Appendix*, Fig. S4).

**Fig. 4.**
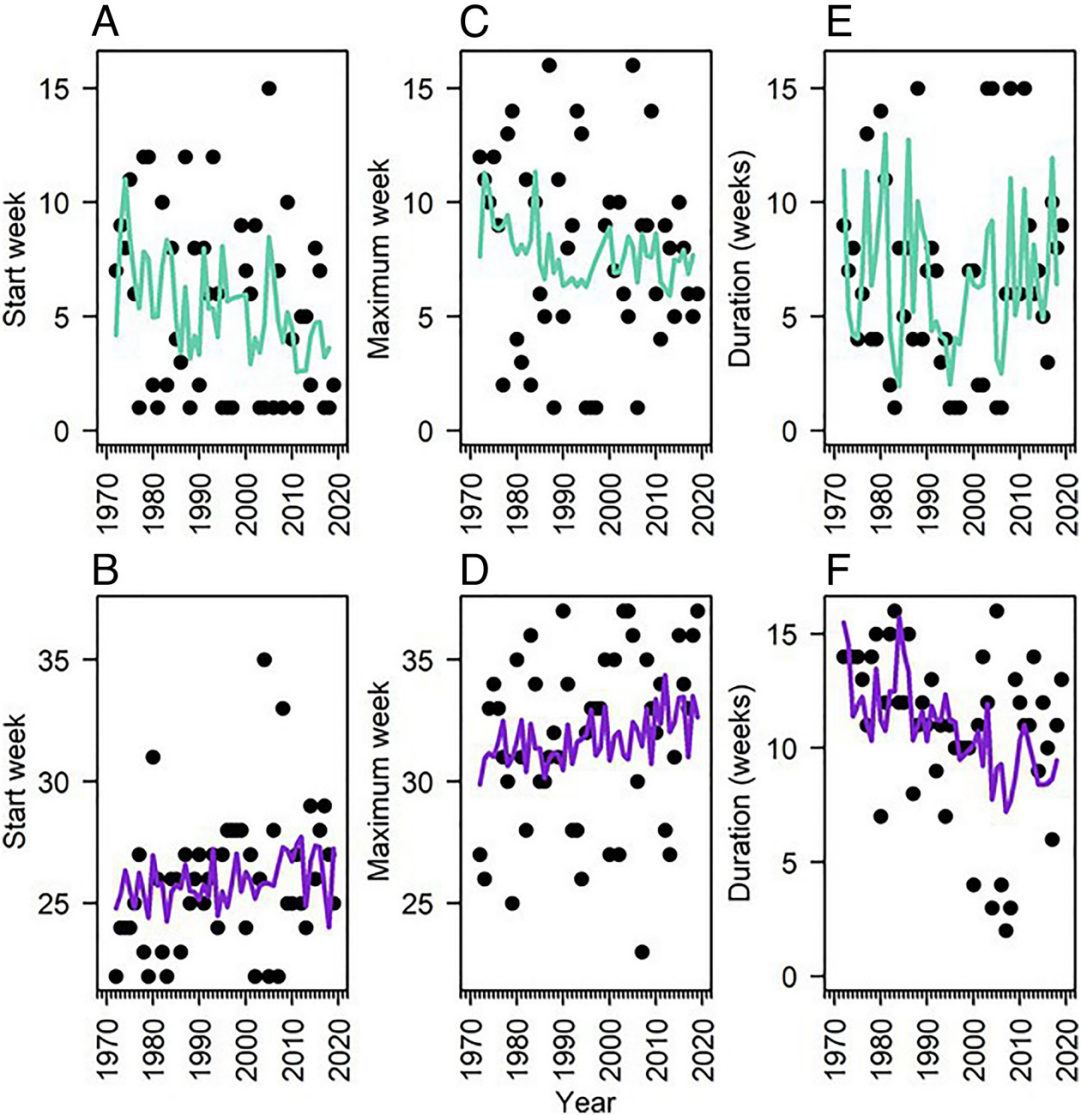
Annual phenology metrics (1970 to 2019) for week of bloom start in winter–spring (*A*) and summer-fall (*B*), week of bloom maximum in winter-spring (*C*) and summer–fall (*D*) and bloom duration in winter-spring (*E*) and summer-fall (*F*). Colored lines indicate generalized linear model (GLM) results with significant environmental predictors used to explain variation for that particular phenology metric. For GLM statistics, refer to *SI Appendix*, Table S4.

Some aspects of the phenology of phytoplankton blooms, identified by thresholds in chl *a* concentration, showed significant temporal shifts. The initiation of the winter-spring bloom occurred earlier in the year (the variable “year” in the Poisson GLM, *SI Appendix*, Table S4). In the first decade of the dataset, the winter–spring bloom began on week 7 ± 4 (mid-Feb) while in the last decade, it began on week 4 ± 3 (late Jan, Fig. 4*A*). The initiation of the summer–fall bloom did not change over time and was variable: the onset ranged from week 22 (early June) to week 35 (late Aug) with a mean initiation of 26 ± 3 wk (late June, Fig. 4*B*). The timing of the bloom maximum did not change for either the winter–spring or summer–fall blooms (*SI Appendix*, Table S4 and Fig. 4 *C* and *D*). On average, the peak of the bloom in winter–spring occurred on week 8 (±4 wk, late Feb/early Mar) and in summer–fall on week 32 (±3 wk, early Aug). The duration of the winter–spring bloom did not change significantly over time and lasted 6 ± 4 wk (*SI Appendix*, Table S4, *P* > 0.05, Fig. 4*E*). In contrast, the duration of the summer–fall bloom decreased significantly over time (*SI Appendix*, Table S4, *P* < 0.001, Fig. 4*F*). Blooms lasted more than ten weeks on average in the first two decades of the time series and less than 10 wk on average in the latter half of the dataset. Bloom frequency varied by season with significantly fewer blooms in winter–spring (median = 3) than in summer–fall (median = 4; *SI Appendix*, Fig. S5) with no long-term, directional trends. The number of blooms throughout the year ranged from three to twelve blooms with a median number of ~seven blooms per year (*SI Appendix*, Fig. S5*C*). There were no significant changes in nutrient phenology (week of nutrient maximum concentration, *P* > 0.05, *SI Appendix*, Fig. S6).

Bloom phenology was associated with multiple environmental factors that varied by season (*SI Appendix*, Table S4). We found that NO_3/2_ concentration, Secchi depth, and salinity were positively related to the timing of both bloom initiation and maximum chl *a* concentration in winter–spring indicating that a shallower Secchi depth as well as lower NO_3/2_ concentrations and salinity corresponded to earlier blooms. Lower SiO_4_ concentrations and less water column stratification were related to an earlier bloom initiation and earlier maximum, respectively. In addition, lower NO_3/2_ and SiO_4_ concentrations explained longer winter–spring bloom durations. Finally, the Gulf Stream Index (GSI) was an important predictor with a positive GSI related to earlier bloom start and maximum as well as a shorter duration. In the summer–fall, no statistically significant environmental parameters explained variation and trends for bloom start or maximum but for bloom duration lower salinity concentrations corresponded to longer blooms (*SI Appendix*, Table S4).

## Discussion

### Long-term Changes in Narragansett Bay.

Phytoplankton-derived chl *a* concentrations in NBay exhibited long-term annual declines from 1968 through 2019, regardless of season, and provide context for reports of summertime declines in NBay chl *a* concentrations (14, 32). Furthermore, the annual maximum chl *a* concentration decreased over time suggesting a waning intensity of phytoplankton bloom events. Long-term declines of chl *a* concentrations in NBay mirror those observed in diverse locations including other estuaries (19, 39), the Gulf of Maine (27), the Australian coast (40), and the Black Sea (41), where blooms have also decreased in intensity over time (41, 42). Reductions in annual and event-scale chl *a* concentrations have both ecological and biogeochemical implications for ecosystem functioning, with decreases in organic matter deposition resulting in a less efficient biological pump (43, 44), and decline in nutrient cycling and benthic production within estuaries (45, 46).

Although the plankton time series provides a rich dataset for examining long-term trends in Narragansett Bay, there are limitations inherent in the study. For example, the weekly sampling frequency may miss the annual chl *a* maximum and cannot capture bloom events shorter than 7 d. Weekly sampling becomes less of an issue as the duration of the time series increases (47), although it may be one reason we observed large variation in the annual chl *a* maximum, which is superimposed on a long-term decrease. Furthermore, chl *a* is a proxy for carbon (C) biomass and changes in chl *a*:C ratios can occur when species composition shifts (48). Future work examining species composition at the time series site may provide insights into the extent to which changes in chl *a*:C have occurred. Finally, single-station time series do not reveal long-term changes in spatial gradients of chlorophyll *a* (49).

At the NBPTS site, the long-term decline of chl *a* concentration was related to an interacting set of environmental factors. To identify predictive causal relationships between time series of chl *a* and environmental data, we used Granger causality analyses. Granger causality determines whether one variable can predict the other, but not the direction of that change (37, 38). Based on this analysis, we found both direct and indirect influences of environmental factors on chl *a*. Nitrate plus nitrite concentrations were primary predictors of chl *a* concentration as expected based on the reliance of phytoplankton growth on these often-limiting nutrients in marine habitats (50). Precipitation was also a primary predictor of chl *a* concentration but likely affected phytoplankton less directly. Freshwater inputs driven by precipitation combined with increasing temperature act to reduce water column mixing, preventing nutrient-rich deep water from reaching the surface to fuel phytoplankton growth (4). This hypothesis is supported by the observation that nutrient concentrations (PO_4_, NO_3/2_, NH_4_, SiO_4_) were Granger-caused by water temperature and water column stratification. Together, these results suggest that nutrient limitation through warming-induced water column stratification could explain chl *a* concentration changes over time.

Both models and observations have pointed to the strong influence of water column stratification on nutrient concentrations in a warming ocean and the potential impacts on ecosystem function, including reduced chl *a* concentrations (22, 51, 52). Reductions in anthropogenic nutrient loading associated with upgrades in wastewater treatment facilities in NBay could further influence the observed relationship between nutrients and phytoplankton biomass (31). However, reduced nutrient loading occurred late in the time series [post 2010 (53)], after much of the long-term chl *a* decline had occurred, suggesting that this was not the primary mechanism driving decreases in chl *a* concentration. Our observation that long-term change in phytoplankton biomass in NBay is likely influenced by environmental factors directly impacted by climate change (e.g., warming temperatures, water column stratification) indicates that it is important to account for regional climate change effects in ecosystem-based management (54).

It is essential to note that chl *a* concentration represents a balance of growth and mortality and many of the environmental factors measured at NBPTS affect phytoplankton growth but not mortality (3, 6). Sources of phytoplankton mortality include predation by zooplankton, ingestion by filter feeders, viral activity, and parasitism (55–58). For example, in NBay, microzooplankton grazing removes an average of 96% of phytoplankton primary production annually (53). Therefore, the loss of chl *a* we observed could be reflective of either lower rates of primary production or higher rates of mortality. Since no long-term time series exists for these measurements in NBay, their influence cannot be directly determined, emphasizing the need for a combination of observational and process-oriented, in situ rate measurements to quantify the causes and effects of phytoplankton variability (59).

### Understanding Phytoplankton Seasonality and Phenology within a Complex Ecosystem.

Seasonal patterns of chl *a* concentration in NBay were characterized by a bimodal distribution throughout the time series with blooms occurring both in winter-spring and in summer–fall, a common feature in northern temperate coastal ecosystems (60). The significant annual peak of chl *a* concentration in winter–spring throughout the time series reveals that while the magnitude of the winter–spring bloom is declining, it continues to be an important component of the annual cycle in NBay. Interestingly, the summer–fall bloom, a significant component of the present-day annual cycle (61), has been characteristic of NBay since the beginning of the chl *a* time series (1968) and may play an increasingly important role in fueling the ecosystem through organic matter production if winter–spring bloom intensity continues to decline.

To further investigate bloom dynamics, we generated a time series of key bloom phenology metrics including the dates of initiation and maximum chl *a* concentration, as well as duration, and frequency. Blooms were defined as time periods exceeding 5% of the annual chl *a* median for each year (62). In NBay, the winter–spring bloom occurred earlier, at a rate of 4.9 ± 2.8 d decade^−1^, which is similar to the rate observed (4.4 ± 0.7 d decade^−1^) in the global ocean (26). This result reveals that the NBay winter-spring bloom may follow similar global patterns of marine phenology, reflecting a strong signal of climate change on phytoplankton bloom initiation from global to regional scales. Given that shifts in the initiation of the winter–spring bloom have yet to be detected elsewhere along the Northeast US Shelf (63), NBay may act as a sentinel ecosystem for monitoring broader phenologic changes across the region.

All other phenology metrics, except summer–fall duration, were highly variable and did not exhibit significant changes over time. For example, the number of blooms occurring annually in NBay was highly variable over time, with bloom frequency ranging from three to twelve blooms per year. Notably, variable phenology is observed in other planktonic ecosystems and is reflective of many interacting abiotic and biotic factors that affect the timing and occurrence of phytoplankton blooms (28).

Seasonal blooms represent short-term events, with increased phytoplankton biomass lasting days to weeks (4). Our SARIMAX model showed that NH_4_, NO_3/2_, and SiO_4_ concentrations were primary predictors of short-term changes in chl *a* concentration, with lower nutrients associated with higher chl *a* concentrations. Similarly, lower NO_3/2_ and SiO_4_ concentrations explained earlier maximum chl *a* concentration during the winter–spring bloom. Lower concentrations of both NO_3/2_ and SiO_4_ were also related to an earlier start and longer duration of the winter–spring bloom. The potentially counterintuitive association between lower nutrients and higher chl *a* concentration or longer blooms is in fact consistent with the well-known relationship of phytoplankton nutrient uptake and resulting growth, such that we expect to see low nutrient concentrations when chl *a* biomass is high (47). Our SARIMAX model results also showed that Secchi depth was associated with short-term changes in chl *a* concentration. Increased biomass in the water column during blooms can decrease water clarity (64) explaining the significant relationship of Secchi depth with chl *a* concentration on a zero-week lag. Notably, there was no long-term trend of increasing Secchi depth with decreasing chl *a* concentration. It is known that Secchi depth and chl *a* are well correlated in open ocean habitats (65) but can become decoupled in estuarine environments due to the prevalence of sediments and detrital matter in the water column (66) likely obscuring any long-term trend due to chl *a* concentration.

Although surface water temperature was not a significant predictor of bloom initiation or duration as has been hypothesized for NBay (14, 67), it may have influenced chl *a* phenology indirectly, via the temporal shifts in water column stratification observed here (*SI Appendix*, Table S1). Even short-term increases in stratification can decrease nutrient availability, a scenario that could intensify as winter surface water temperatures continue to increase over time (29). More directly, the physiological effects of temperature on phytoplankton growth could also influence bloom maxima by promoting the growth of certain taxa over others (68). In NBay, some of the most abundant phytoplankton genera (*Skeletonema* and *Thalassiosira*) are represented by seasonally distinct species assemblages with different thermal niches (69–71). Since chl *a* concentration represents all phytoplankton in a community, future examination of structural and phenologic shifts among taxa is needed in NBay, especially for those taxa that are particularly abundant.

In addition to nutrients and temperature, large-scale oceanographic processes may influence bloom phenology in NBay. For example, a positive GSI [the latitudinal position of the Gulf Stream northern wall (72)] was correlated with later winter-spring *Skeletonema* blooms in NBay, presumably through an increase in water temperature (73). In contrast, we found that for chl *a* concentration, a positive GSI was correlated instead with earlier winter–spring bloom initiation and maximum as well as a shorter duration. Our contrasting results indicate that the whole phytoplankton community, as represented by chl *a* concentration, responds differently to environmental conditions than individual genera. This observation further highlights that chl *a* concentrations represent multiple phytoplankton taxa with unique niches, potentially explaining why one primary driving parameter does not best explain bloom phenology patterns.

**Fig. 5.**
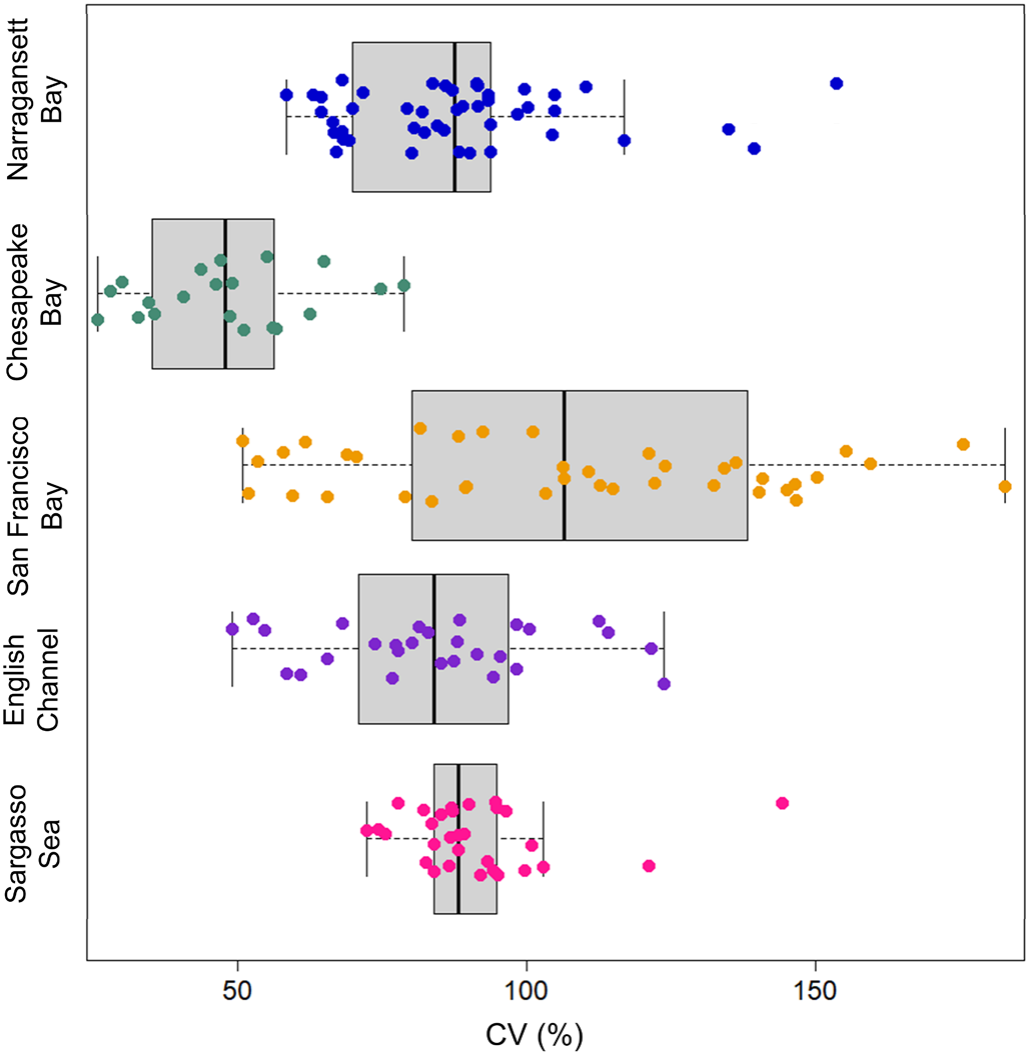
Boxplot illustrating the annual Coeffcient of Variation (CV) for surface chlorophyll *a* (colored circles) from long-term coastal and oceanographic time series. Data points beyond boxplot quantiles represent outliers. Narragansett Bay Long-Term Plankton Time Series (1968 to 2019), Chesapeake Bay (2000 to 2019), San Francisco Bay (1977 to 2015), English Channel, L4 (1992 to 2019), Sargasso Sea, Bermuda Atlantic Time Series (1988 to 2019).

The weekly to decadal-scale variability of chl *a* concentrations in NBay was high, with the annual CV averaging 88% and not significantly changing over time. The consistency of this variation over decades indicates that it may be an inherent characteristic of this estuarine ecosystem. On the one hand, this variability could obscure real temporal trends. On the other hand, the large variability in phytoplankton biomass and some seasonal phenology metrics in spite of increasing water temperatures (30) may point to the resilience of a highly variable system to climate change (74). Interestingly, the variation we observed in NBay (88%) is comparable to that of other marine plankton time series, including those in the oligotrophic open ocean (Fig. 5). In all locations, the average annual CVs were >50%, with a maximum of 182% (San Francisco Bay). The CV at the Bermuda Atlantic Time Series (91%) was similar to NBay and surprising given that oligotrophic regions have much lower chl *a* concentrations, do not experience the large blooms common to coastal and estuarine regions, and thus have lower SD in chl *a* concentrations over time compared to eutrophic regions like NBay (75). When viewed from the normalized perspective of the CV, however, chl *a* time series are highly variable. Thus, the time of emergence for climate change signals in chl *a* across the global ocean is likely on the order of decades (76), highlighting the need to sustain a robust set of marine chl *a* time series for the long term.

## Materials and Methods

Environmental parameters were collected weekly at the NBPTS site from 1959 to 2019 (Table 1 and *SI Appendix*, *Methods*). A substantial component of this study was to digitize physical data archives for weekly surface chl *a* and nutrient concentrations dating from 1959. We compiled newly digitized and previously published data to generate a fully processed, quality-controlled, publicly available dataset for all surface environmental parameters (77).

We applied Bayesian DLMs (e.g., ref. 78) to interpolate missing values in the environmental parameters (Table 1), allowing us to both reconstruct the time series and assess the uncertainty around the missing values. In order to incorporate this uncertainty into our subsequent analysis, we sampled 100 sequences from the posterior predictive distribution of chl *a* and performed a seasonal autoregressive integrated moving average with exogenous variable(s) model (SARIMAX) (79) for each sequence (Table 2). This Monte Carlo approach was used to assess the robustness of the findings under different scenarios of imputed missing values. Granger causality analysis, which identifies predictive causal relationships between two evolving time series, was applied to physical environmental conditions (temperature, nutrient concentrations, stratification, salinity, precipitation, Secchi depth, and light) and chl *a* concentration (37, 38).

Phenology metrics, defined as in Ji et al. (25), included weeks of chl *a* bloom initiation and end, bloom duration, and week of bloom maximum (e.g., peak intensity) for the winter-spring (weeks 1 to 16) and summer-fall (weeks 22 to 38). Bloom start and end weeks were defined with a threshold approach of >5% of the annual chl *a* median for each year (62). To determine environmental parameters related to changes in chl *a* phenology, GLM with a Poisson distribution were used. Additional information on data collection and quality control, DLM and SARIMAX model structures, and environmental parameters used in the GLM can be found in *SI Appendix*, *Methods*.

## Supplementary Material

Appendix 01 (PDF)

## Data Availability

Data for this work are publicly available at the Narragansett Bay Plankton Time Series page on BCO-DMO: https://doi.org/10.26008/1912/bco-dmo.874956.1 (77). Code and associated datasets are publicly available on Github: https://github.com/psthibodeau/Thibodeau-et-al-2023 (80).
