# Supplementary material for: Long-term declines in chlorophyll a and variable phenology revealed by a 60-year estuarine plankton time series
Source: Proc Natl Acad Sci U S A. 2024 May 13;121(21):e2311086121. doi: 10.1073/pnas.2311086121 (PMC11127012; doi:10.1073/pnas.2311086121)
Supplement: Supplementary file 1 — Appendix 01 (PDF) [file pnas.2311086121.sapp.pdf]

PNAS

**Supporting Information for**

# Long-term declines in chlorophyll *a* and variable phenology revealed by a 60-year estuarine plankton time series

Patricia S. Thibodeau, Gavino Puggioni, Jacob Strock, David G. Borkman, Tatiana A. Rynearson

Co-corresponding authors: Tatiana Rynearson, Patricia Thibodeau

Email: rynearson@uri.edu, pthibodeau1@une.edu

**This PDF file includes:**

Supporting text

Figures S1 to S8

Tables S1 to S5

SI References

# SI Appendix

## Supplemental Materials and Methods

### *Study site and data collection*

The Narragansett Bay Long-Term Plankton Time Series (NBPTS) sampling site (41° 34’ 07” N, 71° 23’ 31” W; Fig. 1A) has a depth of 8 m and large annual swings in temperature (< 0°C to > 25°C) (1, 2). It is characterized by a semi-diurnal (12.2h) tidal cycle, which has negligible influence on the long-term and seasonal patterns deduced from measured chlorophyll *a* concentration (chl *a*) (3). The sampling site is relatively well mixed and rarely experiences hypoxia (4). Typical circulation within Narragansett Bay (NBay) occurs with offshore waters entering from Rhode Island Sound into the deeper East Passage of NBay. Water in the upper Bay and East Passage moves west due to prevailing winds and the resulting flow propagates south on the surface in the West Passage where NBPTS is located. Based on examination of current velocities in NBay by Weisberg & Sturges (5) and then Kincaid et al. (6), the subtidal flows that drive NBay estuarine circulation have been relatively stable and velocities seem to generally hold in different areas, like in dredged channels. The range of salinity in NBay is small (22-30) due to the low freshwater input compared to the large tidal volume although estuarine circulation can change with riverine input (7). Previous publicly available chl *a* and nutrient concentration data from 1959-1997 were only available in depth-integrated units over unknown depth intervals. Other environmental parameters from 1959-1997 (e.g., Secchi depth, temperature, salinity, and light) were recorded as discrete surface and depth measurements, previously digitized and made publicly available at: https://www.nabats.org/nabats-data.html. Data (including chl *a* concentration, nutrient concentrations, Secchi depth, temperature, and salinity) from 1999-2019 were recorded as discrete surface and depth measurements and publicly available at: https://web.uri.edu/gso/research/plankton. Data in 2012 were not collected.

Surface water temperature and salinity were determined using a bucket sample, calibrated thermometer (Fischer Scientific) and calibrated refractometer (American Optical) from 1959 to 2008. From 2009-2019, temperature and salinity were determined at 0.5 m depth with a YSI sonde (Model 6920, 6607, or 6659 V2). Total surface chl *a* concentration was determined weekly at the NBPTS location from May 1968-2019 using a bucket sample, by filtering whole

seawater over 25 mm diameter GF/F filters (Whatman, Inc.) and measured fluorometrically using three protocols, either following Yentsch and Menzel (8) as outlined in Li and Smayda (3) after frozen storage at -20°C (1968-1994), or following Strickland and Parsons (9) after frozen storage at -20°C (1997-2007), or following Graff and Rynearson (10) with no filter storage (2008-2019). Compared to immediate extraction, losses of chl *a* concentration following frozen storage at -20°C are significant but predictable, allowing for the use of a correction factor to harmonize the data sets (10). We expanded an existing, corrected chl *a* data set (1999-2007) (10) by applying the same correction factor to historical chl *a* measurements (1968-1994) to account for pigment loss on frozen filters (Table S5). We also examined the uncorrected 1968-1994 dataset, which still indicated a significant decline in chl *a* concentration (Figs. S7 & S8). Note: the correction factor was determined after running the methods described by Li and Smayda (1) and by Graff and Rynearson (5) side by side for an entire year to capture variation across an annual cycle; therefore, we knew chl *a* values measured since 2008 were more accurate because they were determined with immediate extraction (with a high $r^2$ of 92% for comparison of pigment values measured with immediate extraction and frozen storage protocols, Graff & Rynearson (5)). After comparing these data and evaluating the trend in the uncorrected 1968-1994 dataset, we concluded that applying the correction factor to account for pigment lost in frozen extraction was the most appropriate method to handle the full time series presented in this study. The correction factor likely underestimated chl *a* when the large size fraction dominated (5) and thus yielded a conservative adjustment of chl *a*, particularly during the winter-spring bloom.

From 1959-2003, water samples for nutrients were collected with a plastic bucket and stored in 20 L polyethylene carboys until returned to the laboratory for analysis (within 90 minutes) (11) and then filtered through pre-rinsed GFC glass fiber filters (Whatman, Inc.) (12). Between 1969 and 1997, $PO_4$, $SiO_4$, and $NO_3$ were determined via either manual colorimetric methods (13) or automated colorimetric methods using a Technicon autoanalyzer following the methodologies of Furnas et al. (11, 12) and references therein (14–16). $NH_4$ concentrations were determined via the Witting-Buch method (17) from 1972-1980. Post 1980, $NH_4$ measurements were made via automated colorimetric methods using an autoanalyzer following the methodologies of Furnas et al. (12), Furnas (11), and Solóranzo (18). Since only surface $NO_3$

data were available before 1994, and only surface $NO_{3/2}$ data were available in 1995 & 1996, a correction factor of 1.04 was determined through regression analysis by comparing observed $NO_{3/2}$ measurements from 2003-2019 with $NO_3$ concentrations measured during the same period. All available surface data that were collected from 2003-2019 at the time series location when $NO_2$, $NO_3$, and $NO_{3/2}$ samples were analyzed simultaneously ($n = 739$) were used to determine the correction factor ($r = 0.92$, p-value $< 0.001$). Methods of analysis of $PO_4$, $SiO_4$, and $NO_3$ from 1959-1963 were not described in the historic archives but were likely similar to methods used from 1969-1997 (colorimetric). Thus, we present and interpret these earliest measurements (1959-1963) with caution.

From 2003-2019, surface nutrient samples for $PO_4$, $SiO_4$, $NH_4$, and $NO_{3/2}$ were collected weekly at NBPTS and kept on ice and filtered within one to three hours of collection. Filtering was done with acid washed 60 ml syringes and filtering tips (Millipore) using 0.45 μm cellulose filtering membranes (Millipore). Filtrate was placed in 60 ml polyethylene bottles and frozen at -20°C until analysis. Samples were run on a Lachat Quick Chem 8000 Flower Injection Analyzer following Grasshoff (19) for all nutrients as well as and EPA Method 353.4 for $NO_{3/2}$, EPA Method 365.3 for $NH_4$, EPA Method 365.5 and Murphy and Riley (20) for $PO_4$, and Parsons et al. (21) for $SiO_4$. Detection limits for each nutrient are as follows: 0.02 μM for $NO_{3/2}$, 0.05 μM for $NH_4$, 0.01 μM for $PO_4$, and 0.01μM for $SiO_4$. These methods were verified with those from 1969-1997 to ensure continuity throughout the nutrient time series by comparing the references listed within Grasshoff (19), Murphy and Riley (20), and Parsons et al. (21) to those of Strickland and Parsons (13), Furnas et al. (12), and Furnas (11).

Light, measured as incoming atmospheric irradiance (W $m^{-2}$), was collected weekly at NBPTS from 1959 to 1996 and based on the average irradiance during the week (7 days) prior to sampling date. A light sensor was located at either Bunker C (GSO Campus, Narragansett, RI) or at Eppley Labs located in Newport, RI (~10 km east of the NBPTS study site). Photosynthetically active radiation (PAR) data were obtained from the National Estuarine Research Reserve, Narragansett Bay station (41° 38.22' N, 71° 20.34' W) (http://cdmo.baruch.sc.edu/), located ~5 km northeast of the NBPTS study site, to provide light data from 2003 through 2019. A conversion factor (2.1 μmol $m^{-2}$ $s^{-1}$) was used to convert irradiance (W $m^{-2}$) data from 1959-1996 into comparable data in the form of μmol $m^{-2}$ $s^{-1}$ (22).

These data were then aggregated into a weekly value for analysis. Secchi depth (i.e., water clarity) was measured weekly at NBPTS from 1972 to 1996 and then again from Dec 2003 through 2019. Stratification was calculated using the buoyancy frequency (Brunt-Vaisala) with the gsw (Gibbs SeaWater) package v3.06.13 in R (23). The Brunt-Vaisala method has been used to determine stratification in other estuaries including the Chesapeake Bay (24). It requires both surface and bottom salinity and temperature values, which were collected at NBPTS (Surface temperature and salinity data available at https://doi.org/10.26008/1912/bco-dmo.874956.1). Bottom temperature and salinity data publicly available at https://www.nabats.org/nabats-data.html (1959-1997) and https://web.uri.edu/gso/research/plankton (1999-present)).

### *Dynamic Linear Models*

We applied Bayesian Dynamic Linear Models (DLMs; e.g., (25)) to interpolate missing values in the nutrients ($PO_4$, $SiO_4$, $NO_{3/2}$ , $NH_4$), light, salinity (surface and bottom), temperature (surface and bottom), chl *a* concentration, and Secchi depth time series. The datasets exhibited several data gaps, at times up to six years. When available, predictors were extracted from an accompanying nutrient time series ($PO_4$, $SiO_4$, $NH_4$, and $NO_{3/2}$) collected by the Marine Environmental Research Laboratory at University of Rhode Island Graduate School of Oceanography pier (https://web.uri.edu/gso/research/marine-ecosystems-research-laboratory/). Since no data were available from 1963-1969 from either the MERL or NBPTS datasets, the DLM analysis was constricted to 1970-2019.

DLMs are an ideal method for these types of data because they have a highly flexible structure and have the advantage that any parameter evolves with time. Furthermore, since the data are treated as noisy observations of a latent process, the framework is ideal to infer missing data. DLMs utilize two equations: the first is called the observational equation (A) and it models the data $Y_t$ as a realization of an unknown underlying process $\theta_t$ transformed by a known matrix $F_t$ and contaminated by a random noise $v$; the second is called the evolution (or state) equation (B) and it models the time changes in the latent state variable $\theta_t$ which depends in a Markovian fashion on its value at the previous time. Here, the state variable contains the dynamic intercept and slope parameters. The term $w_t$ is called evolution error and determines the stochastic moves from one state to the other.

$$Y_t = F_t\theta_t + v_t, \qquad v \sim N(0, V) \qquad \text{(A)}$$

$$\theta_t = G_t\theta_{t-1} + w_t, \quad w \sim N(0, W) \qquad \text{(B)}$$

The unknown state variables in our case are dynamic intercepts and dynamic regression slopes. Observational and evolutional matrices ($F_t$ and $G_t$) model the structural changes between time periods. Through the structure of these two equations, the DLM is also advantageous in that the latent state provides a smoother representation of the data after subtracting the observational noise. These latent states, conditional on other model parameters, can be solved for in closed form through Kalman Filtering and Smoothing recursions (26). However, because of other unknowns in the model, namely the observational variance ($V$) and evolutionary covariance matrices ($W$), full posterior computation requires simulation methods, typically Markov Chain Monte Carlo (MCMC) algorithms, where each unknown is sampled from its full conditional posterior distribution, a standard technique in Bayesian statistics. To speed up calculations, we used the INLA (Integrated Nested Laplace Approximation) routines, which yield a close approximation to the posterior distribution of the unknowns in these models (27). The dynamic linear model fits had latent state components capturing non-linear trends via a dynamic intercept and changing seasonal cycles via Fourier form frequencies to model cyclic behavior.

The posterior mean of the DLM structural components was used to analyze patterns over time. Modeled chl *a* data were also averaged for each week of each decade to determine significant differences in decadal distributions using the K-sample Anderson-Darling test (28) with Bonferroni p-value adjustment for multiple comparisons. The average cumulative decadal chl *a* value was determined to compare the percent decline in chl *a* concentration in the last decade (2009-2019, not including 2012) to the first complete decade when there were < 10 missing chl *a* values (1973-1982). A percent decline was determined by subtracting the last cumulative decadal average from the first cumulative decadal average and dividing by the first cumulative decadal average. The variance around this value was computed by determining the mean plus standard deviation of the first cumulative decade and the mean minus standard deviation of the last cumulative decade, and vice versa, and then computing the percent decline using the same method described above. A two-sample Student's *t*-test with an $\alpha$ of 0.05 was used to test for significant differences in the mean cumulative sum chl *a* concentrations between the first and last decade. The same method was followed to determine the percent decline in

average maximum annual chl *a* concentration from the first to last decade. Finally, changes in other environmental parameters were determined by comparing the mean intercept for each dynamic linear model for the first (1970-1979) versus last decade (2010-2019) with a two-sample Student's *t*-test. The first decade for light was from 1975-1984 due to extraneous values from 1970-1974. All statistical analyses were conducted in R Version 4.1.2 (29).

### *Phenology metrics*

Raw total surface chl *a* concentration with the posterior predictive mean from the chl *a* DLM were used to impute missing values from 1968 to 2019 to determine phenology metrics. This approach improved detection of bloom phenology metrics by retaining extremes in chl *a* values, which are removed in DLMs. Since most years exhibited a bimodal seasonal cycle, phenology metrics were defined both for blooms in the winter/spring (Jan to Apr, weeks 1 thru 16) and summer-fall (June to Sept, weeks 22 thru 38). Bloom frequency (number of blooms) per annual cycle was also determined for the winter-spring and summer-fall by identifying the number of times chl *a* concentration crossed the > 5% annual chl *a* median each year and in both seasons.

### *Environmental parameters and times series analysis*

Statistical relationships of trends in chl *a* concentrations and bloom phenology with environmental parameters were examined. Environmental parameters included nutrient concentrations, temperature, salinity, stratification, precipitation, light, and Secchi depth collected at the NBPTS location and weekly precipitation collected at the Theodore Francis Green Memorial State Airport in Warwick, Rhode Island (obtained from the NOAA National Centers for Environmental Information, www.ncdc.noaa.gov).

To identify environmental predictors associated with trends in chl *a* concentration, a seasonal autoregressive integrated moving average with exogenous variable(s) model (SARIMAX) was designed (30). This model was used because it accounts for autocorrelation structure common within time series. Models that do not account for this may have independence assumptions that are violated, resulting in misinterpretation (31, 32). A SARIMAX specification models the current value of the observed time series $Y_t$ by using its lagged values and the lagged

values of the disturbance term $\epsilon_t$ to represent short-term variability, seasonal terms for the cyclical components, an integrated term for non-stationary long-term components, and finally a regression component to include exogenous variables with lags as predictors. Identification of the different components was performed using the classical Box-Jenkins identification steps to leave residuals without any significant autocorrelation (30). External predictors were selected by using forward and backward selection procedures to remove statistically non-significant terms and by minimizing the Akaike Information Criterion.

To determine environmental parameters related to changes in chl *a* phenology, generalized linear models (GLM) with a Poisson distribution were used. To ensure consistency in environmental parameters with the phenology metrics, environmental data were aggregated over the same weeks and seasons as the phenology data described above. Since the phenology data were at seasonal intervals, we also included the North Atlantic Oscillation (NAO), Gulf Stream Index (GSI), and the Regional Slope Water Temperature (RSWT) index in the GLMs. The GSI is based on the position of the north wall of the Gulf Stream as it turns eastward, away from the coast of North America (33, 34). Oscillations in the GSI were previously linked to shifts in phytoplankton species abundance in NBay (35). We used the NAO index of Hurrell (36) from https://climatedataguide.ucar.edu/climate-data/hurrell-north-atlantic-oscillation-nao-index-station-based and the GSI index from http://www.pml-gulfstream.org.uk/data.htm.The Regional Slope Water Temperature index is derived from a principal components analysis of eight slope water temperature anomaly time series from the Northwest Atlantic as described in Pershing et al. (37).

### *Phytoplankton time series comparison*

The annual coefficient of variation (CV) for chl *a* concentrations was determined for NBPTS as well as comparable chl *a* time series based on times series length and location. The datasets for the other time series were collected as follows: Chesapeake Bay time series (2000-2019): https://www.chesapeakebay.net/what/downloads/cbp-water-quality-database-1984-present; San Francisco Bay (1977-2015): https://sfbay.wr.usgs.gov/water-quality-database/, L4 Time Series located in the Western English Channel (1992-2019), by request at:

https://www.westernchannelobservatory.org.uk/data.php, BATS: Bermuda Atlantic Time Series (1988-2019): http://bats.bios.edu/bats-data/.

## Supplemental Figures and Tables

### *Figures*

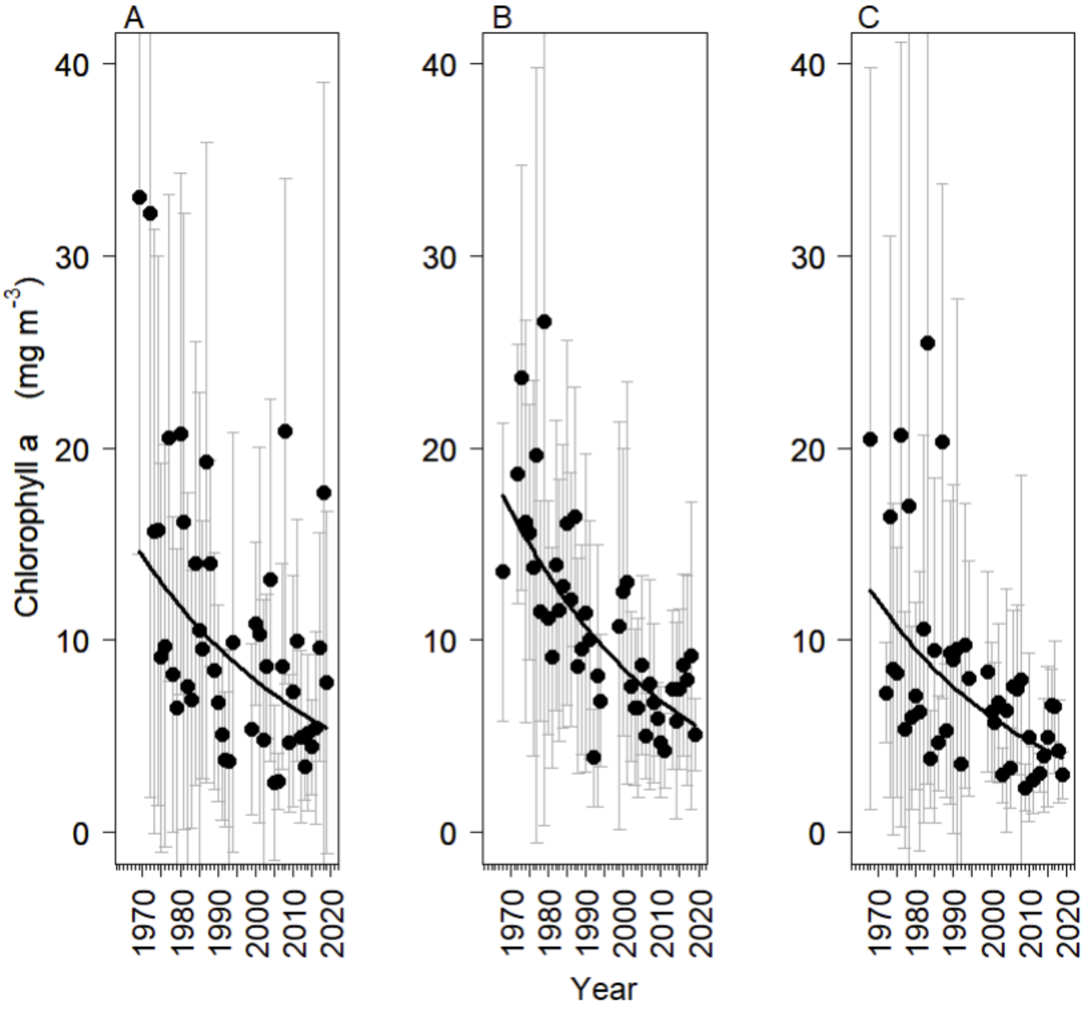


**Figure S1.** Annual mean chlorophyll *a* concentrations by season. Error bars represent one standard deviation. A) winter-spring (Jan to Apr, weeks 1 thru 16), B) summer-fall (June to Sept, weeks 22 thru 38), and C) fall-winter (Oct to Dec, weeks 39 through 52). Regression statistics are as follows: A) $\log((x)) = -0.02x + 41.78$, $R^2 = 0.23$, $p < 0.001$, B) $\log((x)) = -0.02x + 46.98$, $R^2 = 0.53$; $p < 0.001$, C) $\log((x)) = -0.02x + 47.87$, $R^2 = 0.35$; $p < 0.001$.

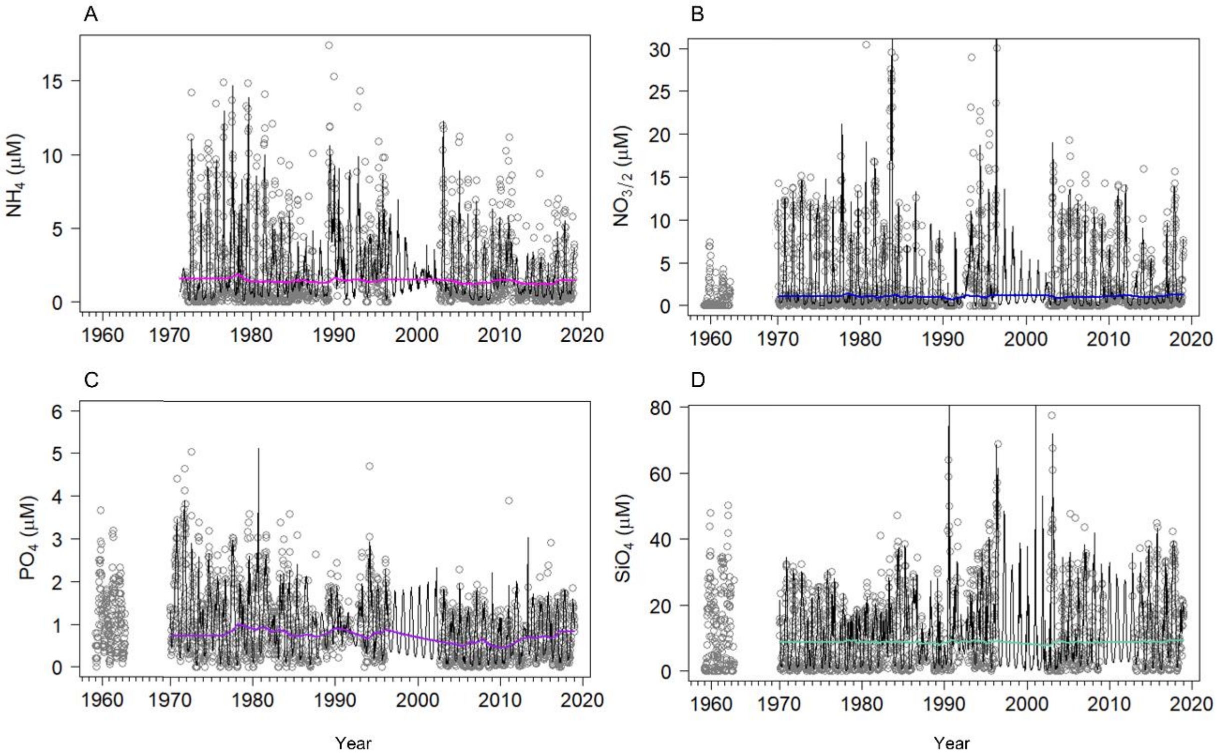


**Figure S2.** Weekly observations of nutrient concentrations (grey circles) measured at the Narragansett Bay Long-Term Plankton Time Series with dynamic linear model (black line) and long-term component (colored line) of the model. A) ammonium ($NH_4$), B) nitrate/nitrite ($NO_{3/2}$), C) phosphate ($PO_4$), and D) silicate ($SiO_4$). Note: One data point removed from B) (1996, 43) and one data point removed from C) (1982, 10) to better illustrate long-term trend.

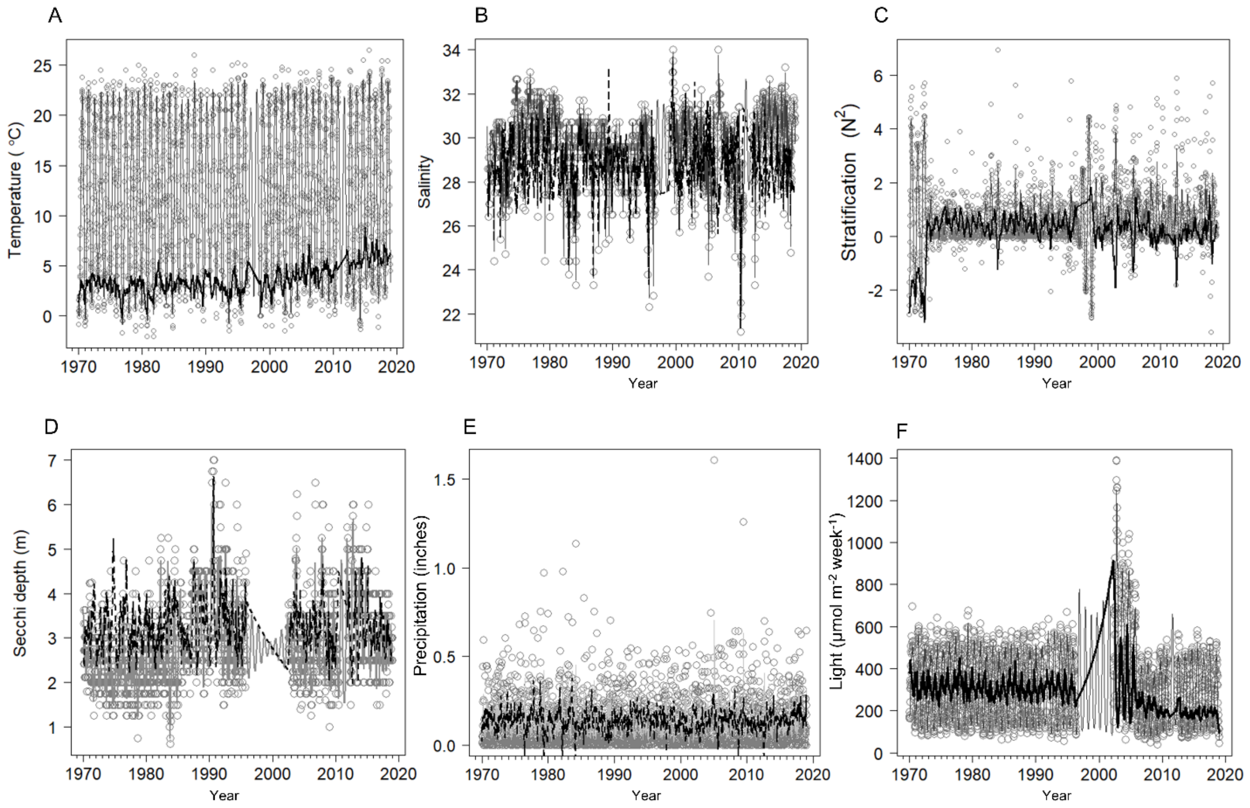


**Figure S3.** Weekly observations (circles) of environmental parameters measured at the Narragansett Bay Long-Term Plankton Time Series with dynamic linear model (grey line) and long-term component (black line) of the model.

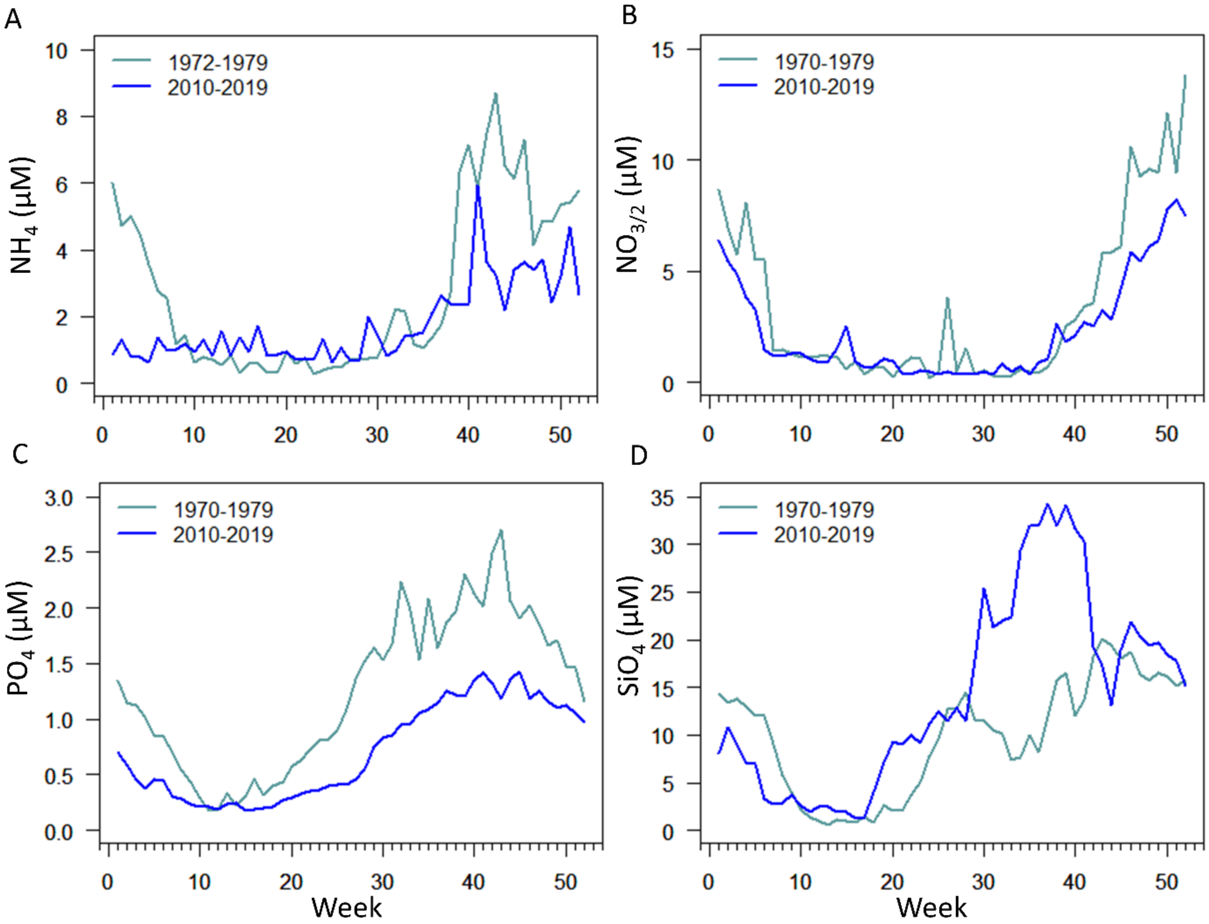


**Figure S4.** Decadal patterns of observed weekly nutrient concentrations based on average concentrations for A) ammonium ($NH_4$), B) nitrate/nitrite ($NO_{3/2}$), C) phosphate ($PO_4$), and D) silicate ($SiO_4$). The range of standard deviations for each nutrient concentration are as follows; 1970-1979 $PO_4$: 0.14-1.29, $SiO_4$: 0.37-10.72, $NO_{3/2}$: 0.11-10.19, $NH_4$: 0.26-5.81; 2010-2019 $PO_4$: 0.08-1.19, $SiO_4$: 0.69-17.63, $NO_{3/2}$: 0.26-5.04, $NH_4$: 0.46-3.24 µM. Note: Observed $NH_4$ data are not available in 1970 & 1971.

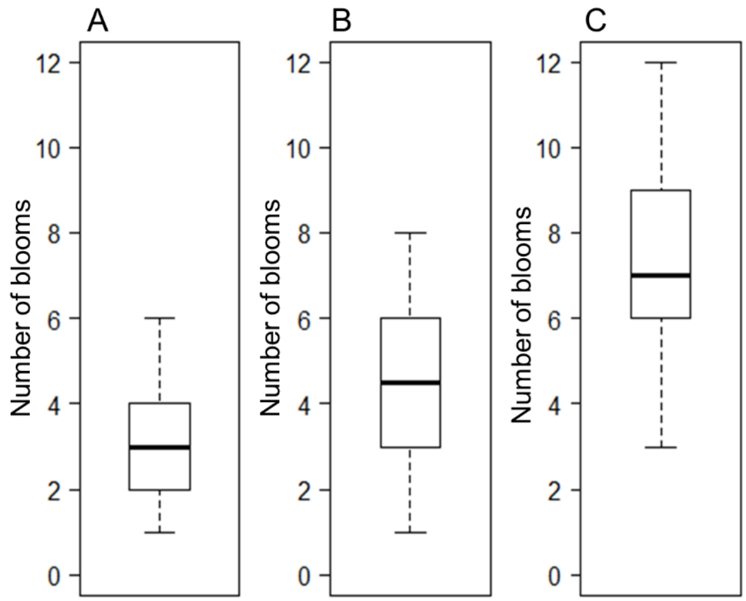


**Figure S5.** Boxplot of bloom frequency (number of blooms) by season, A) winter-spring, B) summer-fall, and C) for the full year.

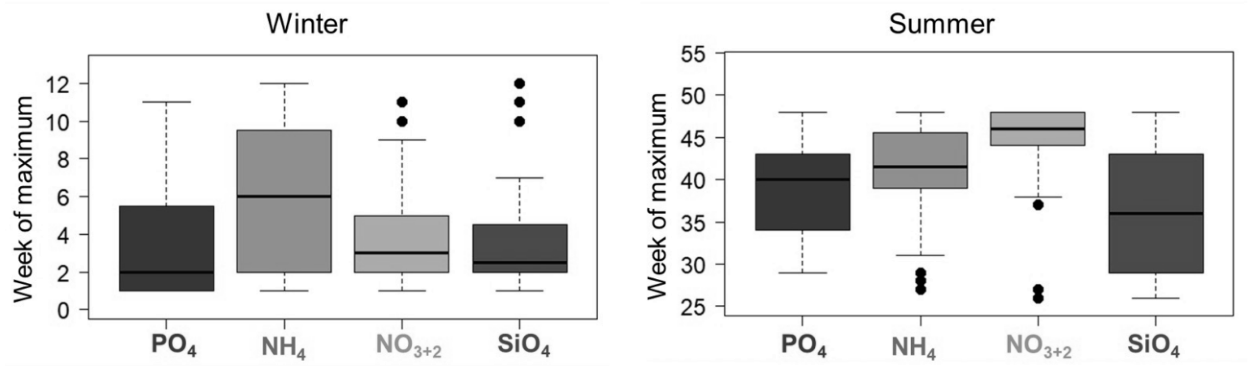


**Figure S6.** Nutrient phenology as indicated by week of nutrient maximum concentration in winter-spring and summer-fall. Boxplots represent seasonal median concentrations (solid black line) with 25 and 75% quantile concentrations. Dots indicate outliers. Nutrients are presented left to right in µM. No long-term, directional trend in the timing of the maximum nutrient concentration for any nutrient ($p > 0.05$).

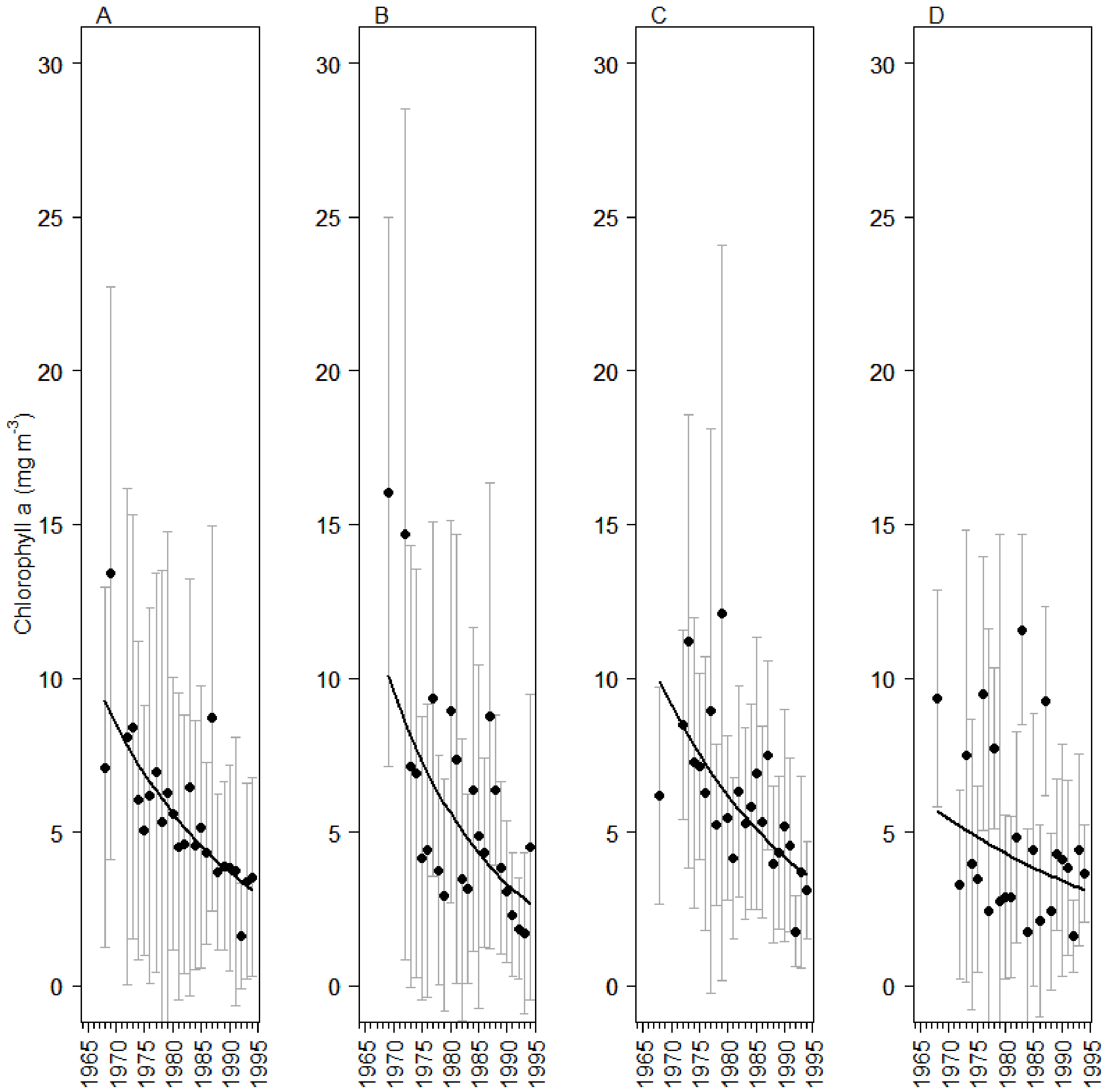


**Figure S7.** Annual mean uncorrected chlorophyll *a* concentrations (1968-1994) by year (A) and season (B-D). Error bars represent one standard deviation. B) winter-spring (Jan to Apr, weeks 1 thru 16), C) summer-fall (June to Sept, weeks 22 thru 38), and D) fall-winter (Oct to Dec, weeks 39 through 52). Regression statistics are as follows: A) log((x)) = -0.04x + 84.06, $R^2$ = 0.57, $p < 0.001$, B) log((x)) = -0.05x + 106.61, $R^2$ = 0.40, $p < 0.001$, C) log((x)) = -0.03x + 78.82, $R^2$ = 0.44; $p < 0.001$, D) log((x)) = -0.02x + 47.14, $R^2$ = 0.05; $p = 0.14$.

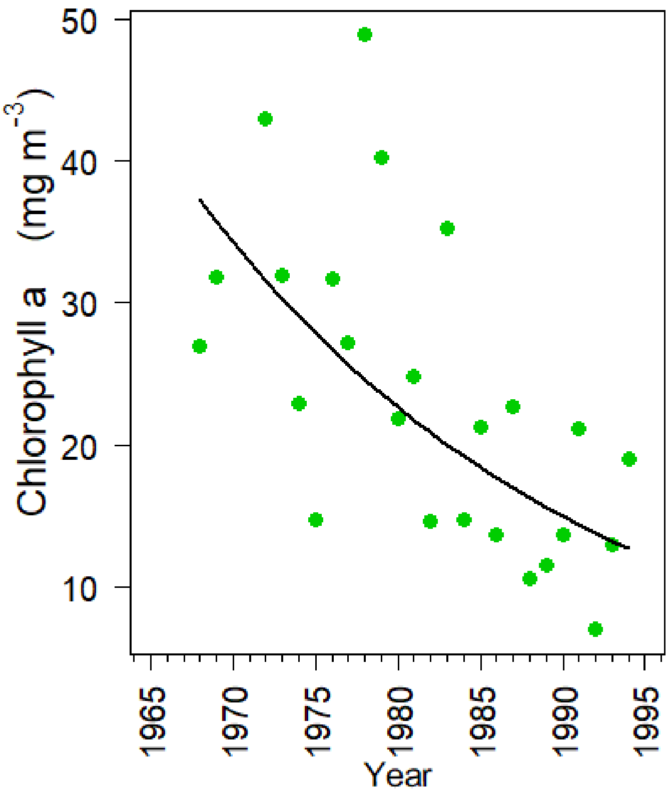


**Figure S8.** Annual maximum uncorrected chlorophyll *a* concentrations (1968-1994) with the log-transformed model fit. Regression statistics are as follows: log((x)) = -0.04x + 84.92, $R^2 = 0.40$, $p < 0.001$.

*Tables*

**Table S1.** Environmental parameters recorded from the Narragansett Bay Long-Term Plankton Time Series (*see* Table 1 for years sampled) presented as ranges for observed data and by the mean ± standard deviation of the first (1970-1979) versus last decade (2010-2019) of values based on the intercept for each dynamic linear model. Temp – Temperature (°C), Strat – Stratification, Secchi – Secchi depth, $PO_4$ – phosphate, $NH_4$ – ammonium, $NO_{3/2}$ – nitrate/nitrite, $SiO_4$ – silicate, Chl *a* – Chlorophyll *a*, Bd – below detection, t – t-value, p – p-value from Student's t-test. The p-value is the results of testing for differences between values in the first and last decades. Note: first decade for Light from 1975-1984 due to extraneous values from 1970-1974 (*see* Fig. S3F) and no data were collected in 2012 for any parameter.

| Parameter | Range | 1970-1979 | 2010-2019 | t | p |
|---|---|---|---|---|---|
| **Chl *a*** (mg $m^{-3}$) | 0.05 – 107 | 7.79 (0.59) | 5.58 (0.31) | 74.86 | < 0.001 |
| **$NH_4$** (µM) | bd – 17.45 | 1.58 (0.08) | 1.34 (0.11) | 38.48 | < 0.001 |
| **$NO_{3/2}$** (µM) | bd – 43.49 | 1.17 (0.06) | 1.14 (0.08) | 7.33 | < 0.001 |
| **$PO_4$** (µM) | bd – 10.35 | 0.79 (0.08) | 0.66 (0.12) | 20.11 | < 0.001 |
| **$SiO_4$** (µM) | bd – 77.62 | 8.85 (0.16) | 8.87 (0.19) | -1.57 | > 0.05 |
| **Temp** (°C) | -2 – 26.47 | 2.94 (0.81) | 5.57 (0.89) | -49.68 | < 0.001 |
| **Salinity** | 3.8 – 34 | 28.77 (1.01) | 28.79 (1.31) | -0.26 | > 0.05 |
| **Strat** ($N^2$) | -8.03 – 5.05 | 0.43 (0.36) | 0.04 (0.38) | 17.05 | < 0.001 |
| **Secchi** (m) | 0 – 35 | 3.23 (0.54) | 3.27 (0.57) | -1.22 | > 0.05 |
| **Precip** (in) | 0 – 1.61 | 0.14 (0.05) | 0.14 (0.04) | 1.58 | > 0.05 |
| **Light** (µmol $m^{-2}$ $w^{-1}$) | 41.60 – 3486.18 | 317.94 (43.27) | 190.27 (19.97) | 61.08 | < 0.001 |

**Table S2.** Comparisons of chlorophyll *a* (chl *a*) by annual coefficient of variation (CV%), cumulative annual chl *a* value (mean ± standard deviation, mg $m^{-3}$), and annual maximum chl *a* value (mean ± sd, mg $m^{-3}$) for the first complete decade (1973-1982) and last complete decade (2009-2019). Significant differences between mean decadal values determined with a Student's t-test ($p < 0.05$).

| Variable | First decade | Last decade | p-value |
|---|---|---|---|
| **Average annual CV** | 99.92 ± 26.02 | 83.42 ± 22.78 | 0.15 |
| **Cumulative annual chl *a*** | 574.92 ± 131.30 | 290.16 ± 93.26 | < 0.001 |
| **Average annual max chl *a*** | 59.90 ± 23.38 | 25.63 ± 13.90 | 0.001 |

**Table S3.** Pairwise comparisons of weekly chlorophyll *a* (chl *a*) concentration distribution averaged by decade using the K-sample Anderson-Darling All-Pairs Test with Bonferroni p-adjusted values. Adjusted p-values indicated as follows: * $p < 0.05$, ** $p < 0.01$, *** $p < 0.001$.

| **Parameter** | | 1970s | 1980s | 1990s | 2000s |
|---|---|---|---|---|---|
| **Chl *a*** | 1980s | * | | | |
| | 1990s | *** | *** | | |
| | 2000s | *** | *** | * | |
| | 2010s | *** | *** | | ** |

**Table S4**. Poisson generalized linear model results explaining long-term changes of chlorophyll *a* phenology (week of bloom start/initiation, maximum, and duration) by season (winter-spring and summer-fall). Model component abbreviations are as follows: Chl – Chlorophyll *a*, NAO – North Atlantic Oscillation, GSI – Gulf Stream Index, RSWT – Regional Slope Water Temperature, Secchi – Secchi depth, $PO_4$ – phosphate, $NH_4$ – ammonium, $SiO_4$ – silicate, $NO_{3/2}$ – nitrate/nitrite.

| | *Winter-Spring* | | | | | *Summer-Fall* | | | | |
|---|---|---|---|---|---|---|---|---|---|---|
| | **Parameter** | **Estimate** | **Std error** | **z value** | **p** | **Parameter** | **Estimate** | **Std error** | **z value** | **p** |
| *Start* | ***Year*** | -0.013 | 0.006 | -2.281 | 0.022 | ***NAO*** | -0.046 | 0.037 | -1.247 | 0.213 |
| | ***GSI*** | -0.242 | 0.106 | -2.285 | 0.022 | | | | | |
| | ***Secchi*** | 0.565 | 0.120 | 4.712 | < 0.001 | | | | | |
| | ***$NO_{3/2}$*** | 0.096 | 0.029 | 3.286 | 0.001 | | | | | |
| | ***$SiO_4$*** | 0.042 | 0.017 | 2.466 | 0.013 | | | | | |
| | ***Salinity*** | 0.264 | 0.076 | 3.459 | < 0.001 | | | | | |
| *Max* | ***GSI*** | -0.309 | 0.087 | -3.541 | < 0.001 | ***Temperature*** | 0.054 | 0.044 | 1.222 | 0.222 |
| | ***Secchi*** | 0.463 | 0.094 | 4.924 | < 0.001 | | | | | |
| | ***Stratification*** | 0.256 | 0.181 | 1.413 | 0.157 | | | | | |
| | ***$NO_{3/2}$*** | 0.062 | 0.023 | 2.612 | 0.008 | | | | | |
| | ***Salinity*** | 0.166 | 0.056 | 2.940 | 0.003 | | | | | |
| *Duration* | ***GSI*** | -0.277 | 0.079 | -3.506 | < 0.001 | ***Year*** | -0.013 | 0.003 | -3.631 | < 0.001 |
| | ***$SiO_4$*** | -0.054 | 0.017 | -3.208 | < 0.001 | ***RSWT*** | 0.024 | 0.027 | 0.859 | 0.390 |
| | ***$NO_{3/2}$*** | -0.123 | 0.033 | -3.648 | < 0.001 | ***$SiO_4$*** | 0.013 | 0.009 | 1.425 | 0.154 |
| | | | | | | ***Salinity*** | -0.087 | 0.044 | -1.978 | 0.047 |

**Table S5.** Summarized chronology of chlorophyll *a* extraction and filter storage methods and the correction factor used (if applicable) for the Narragansett Bay Long-Term Plankton Time Series surface water chlorophyll *a* dataset (1968-2019). Note: no chlorophyll *a* data were collected in 1995 and 1996.

| Time period | Extraction Method | Filter Storage | Correction factor |
|---|---|---|---|
| 2008-2019 | Graff & Rynearson (2011) with Strickland & Parsons (1972) | No freezing, immediate extraction | None |
| 1997-2007 | Strickland & Parsons (1972) | Frozen storage at -20°C | 1.45 |
| 1968-1994 | Yentsch & Menzel (1963) | Frozen storage at -20°C | 1.45 |

## SI References


1. S. W. Nixon, *et al.*, The impact of changing climate on phenology, productivity, and benthic–pelagic coupling in Narragansett Bay. *Estuarine, Coastal and Shelf Science* **82**, 1–18 (2009).

2. R. W. Fulweiler, A. J. Oczkowski, K. M. Miller, C. A. Oviatt, M. E. Q. Pilson, Whole truths vs. half truths – And a search for clarity in long-term water temperature records. *Estuarine, Coastal and Shelf Science* **157**, A1–A6 (2015).

3. Y. Li, T. J. Smayda, Temporal variability of chlorophyll in Narragansett Bay, 1973-1990. *ICES Journal of Marine Science* **55**, 661–667 (1998).

4. C. Oviatt, *et al.*, Managed nutrient reduction impacts on nutrient concentrations, water clarity, primary production, and hypoxia in a north temperate estuary. *Estuarine, Coastal and Shelf Science* **199**, 25–34 (2017).

5. R. H. Weisberg, W. Sturges, Velocity observations in the West Passage of Narragansett Bay: A Partially Mixed Estuary. *Journal of Physical Oceanography* **6**, 345–354 (1976).

6. C. Kincaid, L. Bergondo, D. Rosenberger, "The dynamics of water exchange between Narragansett Bay and Rhode Island Sound" in *Science for Ecosystem-Based Management*, (Springer, 2008), pp. 301–324.

7. L. Bergondo, "Examining the processes controlling water column variability in Narragansett Bay: Time series data and numerical modeling," University of Rhode Island. (2004).

8. C. S. Yentsch, D. Menzel, A method for the determination of phytoplankton chlorophyll and phaeophytin by fluorescence. *Deep-Sea Research* **10**, 221–231 (1963).

9. J. D. H. Strickland, T. R. Parsons, *A practical handbook of seawater analysis* (Fisheries Research Board of Canada, 1972).

10. J. R. Graff, T. A. Rynearson, Extraction method influences the recovery of phytoplankton pigments from natural assemblages. *Limnology and Oceanography: Methods* **9**, 129–139 (2011).

11. M. J. Furnas, Nitrogen dynamics in lower Narragansett Bay, Rhode Island. I. Uptake by size-fractionated phytoplankton populations. *Journal of Plankton Research* **5**, 657–676 (1983).

12. M. J. Furnas, G. L. Hitchcock, T. J. Smayda, Nutrient-Phytoplankton Relationships in Narragansett Bay During the 1974 Summer Bloom. *Estuarine Processes*, 118–133 (1976).

13. J. D. H. Strickland, T. R. Parsons, *A manual of seawater analysis*, 2nd Ed. (Fisheries Research Board of Canada, 1965).

14. F. A. J. Armstrong, The determination of silicate in seawater. *Journal of Marine Biology Association UK* **30**, 149–160 (1951).

15. K. Grasshoff, "Automatic determination of fluoride, phosphate and silicate in seawater" in *Automation in Analytic Chemistry*, L. T. Skiggs Jr., Ed. (Mediad, Inc., 1966), pp. 304–307.

16. E. D. Wood, F. A. J. Armstrong, F. A. Richards, Determination of nitrate in seawater by cadmium-copper reduction. *Journal of Marine Biology Association UK* **47**, 23–31 (1967).

17. I. Barnes, Apparatus and methods of oceanography. Part one: Chemical. *Allen and Unwin* (1959).

18. L. Solorzano, Determination of ammonia in natural waters by the phenylhypochlorite method. *Limnology and Oceanography* **14**, 799–801 (1969).

19. K. Grasshoff, *Methods of Seawater Analysis*, 2nd Ed. (Verlag Chemie, 1976).

20. J. Murphy, J. P. Riley, A modified single solution method for the determination of phosphate in natural waters. *Analytica Chimica Acta* **27**, 31–36 (1962).

21. T. Parsons, Y. Maita, C. Lalli, *A manual of chemical and biological methods for seawater analysis* (Pergamon Press, 1984).

22. J. C. Sager, C. McFarlane, "Radiation" in *Growth Chamber Handbook*, R. W. Langhans, T. W. Tibbitts, Eds. (Iowa State University, 1997), pp. 1–30.

23. T. McDougall, B. Barker, Buoyancy (Brunt-Vaisala) frequency squared (N2) (75-term equation) (2021).

24. W. M. Kemp, *et al.*, Eutrophication of Chesapeake Bay: Historical trends and ecological interactions. *Marine Ecology Progress Series* **303**, 1–29 (2005).

25. M. West, J. Harrison, *Bayesian Forecasting and Dynamic Models*, 2nd Ed. (Springer-Verlag, 1997) https:/doi.org/10.1007/b98971.

26. R. E. Kalman, A New Approach to Linear Filtering and Prediction Problems. *Transactions of the ASME Journal of Basic Engineering* **82**, 35–45 (1960).

27. S. Martino, A. Riebler, Integrated Nested Laplace Approximations (2020) https:/doi.org/https://doi.org/10.1002/9781118445112.stat08212.

28. F. Scholz, M. A. Stephens, K-Sample Anderson–Darling Tests. *Journal of the American Statistical Association* **82**, 918–924 (1987).

29. R Core Team, R: A language and environment for statistical computing (2021).

30. J. D. Cryer, K. Chan, *Time Series Analysis with applications in R*, 2nd Ed. (Springer, 2008).

31. G. U. Yule, Why Do We Sometimes Get Nonsense Correlations between Time Series? A Study in Sampling and the Nature of Time Series. *Journal Of The Royal Statistical Society* **89**, 1–64 (1926).

32. R. T. Dean, W. T. M. Dunsmuir, Dangers and uses of cross-correlation in analyzing time series in perception, performance, movement, and neuroscience: The importance of constructing transfer function autoregressive models. *Behavior research methods* **48**, 783–802 (2016).

33. A. H. Taylor, North-south shifts in the Gulf Stream and their climatic connections with the abundance of zooplankton in the U.K. and its surrounding seas. *ICES Journal of Marine Science* **52**, 711–721 (1995).

34. A. H. Taylor, North-south shifts in the Gulf Stream: ocean-atmosphere interactions in the North Atlantic. *International Journal of Climatology* **16**, 559–583 (1996).

35. D. G. Borkman, T. Smayda, Multidecadal (1959-1997) changes in *Skeletonema* abundance and seasonal bloom patterns in Narragansett Bay, Rhode Island, USA. *Journal of Sea Research* **61**, 84–94 (2009).

36. J. W. Hurrell, Decadal trends in the North Atlantic oscillation. *Science* **269**, 676–679 (1995).

37. A. J. Pershing, C. H. Greene, C. Hannah, D. Sameoto, Oceanographic responses to climate in the Northwest Atlantic. *Oceanography* **14**, 76–82 (2001).
